# A case of complete remission of multiple lung metastases following surgical resection of huge hepatocellular carcinoma invading the colon and postoperative treatment with atezolizumab and bevacizumab

**DOI:** 10.1007/s12328-026-02360-x

**Published:** 2026-05-18

**Authors:** Mayuko Ohara, Yoji Kishi, Hanae Shinada, Kazuki Kobayashi, Naoto Yonamine, Takazumi Tsunenari, Mikiya Takao, Takahiro Einama, Hideki Ueno

**Affiliations:** Department of Surgery, National Defense Medical College, 3-2 Namiki, Tokorozawa, Saitama 359-8513 Japan

**Keywords:** Hepatocellular carcinoma, Colon invasion, Lung metastases, Atezolizumab and bevacizumab

## Abstract

We report a rare case of giant hepatocellular carcinoma (HCC) with direct invasion to the colon and synchronous multiple lung metastases that achieved complete remission with surgical hepatectomy followed by chemotherapy with atezolizumab and bevacizumab. An 80-year-old man presented with abdominal distension and appetite loss. Imaging studies demonstrated a large HCC protruding from the liver with compression and invasion of the ascending colon, accompanied by multiple bilateral pulmonary nodules. To relieve symptoms and reduce the risk of tumor rupture, upfront surgical resection consisting of right hepatectomy and right hemicolectomy was performed. Histopathological examination revealed moderately to poorly differentiated HCC with direct invasion into the ascending colon and microscopic portal vein invasion. Postoperatively, tumor markers initially decreased; however, subsequent elevation led to initiation of atezolizumab plus bevacizumab for treatment of pulmonary metastases. Tumor markers subsequently normalized, and follow-up computed tomography demonstrated disappearance of all pulmonary metastases, with only scar-like lesions remaining. Radiologic complete remission was achieved 14 months after hepatectomy. The patient remains alive without recurrence 54 months after surgery. Direct invasion of HCC into adjacent organs is extremely rare and is generally associated with poor prognosis. The role of systemic chemotherapy after reduction surgery for advanced HCC remains controversial. Although this is a single case experience and caution is needed when generalizing, this case suggested that combined treatment with initial resection of the primary tumor followed by immunotherapy for remnant lesions could be feasible in selected patients and would potentially contribute to symptom relief and control of metastatic lesions.

## Introduction

According to the current Japanese guidelines [1], combination immunotherapy with atezolizumab plus bevacizumab is the first-line treatment for patients with unresectable hepatocellular carcinoma (HCC) and Child–Pugh class A liver function, as this regimen has been shown to provide superior overall and progression-free survival compared with sorafenib [2]. Previous studies reported response rate, tumor control rate, and progressive disease rate of 30%, 74%, and 19%, respectively [3, 4]. Atezolizumab is an immune checkpoint inhibitor that binds to the cell surface protein PD-L1, promoting immune cell attack [5]. Bevacizumab is a vascular endothelial growth factor inhibitor that suppresses tumor angiogenesis [6]. Treatment with these agents is associated with marked tumor responses and in selected patients with initially unresectable disease conversion to resectable disease has been achieved. The RACB study was a multicenter, prospective, single-arm phase 2 trial conducted in Japan to evaluate conversion surgery using atezolizumab plus bevacizumab in patients with technically or oncologically unresectable HCC. The study reported a complete response rate of 2.2% and a partial response rate of 26.1% based on mRECIST criteria [7], with an overall resection rate of 48%. Nevertheless, reports of the utility of this combination therapy as part of multidisciplinary treatment after surgical resection are rare [8].

The most frequent pattern of metastatic spread in HCC is intrahepatic metastasis via the intrahepatic portal veins [9]. In cases with extrahepatic spread, HCC commonly metastasizes via the lymphatic system or bloodstream, most frequently to the lungs, lymph nodes, and bones [10, 11]. Direct invasion to the adjacent organs is rare, with only a few cases having been reported in the literature [12–14].

Here, we report a patient with a giant HCC invading the ascending colon and multiple pulmonary metastases who was successfully treated with surgical resection of the primary lesion followed by combination therapy with atezolizumab and bevacizumab.

### Case report

**Fig. 1:**
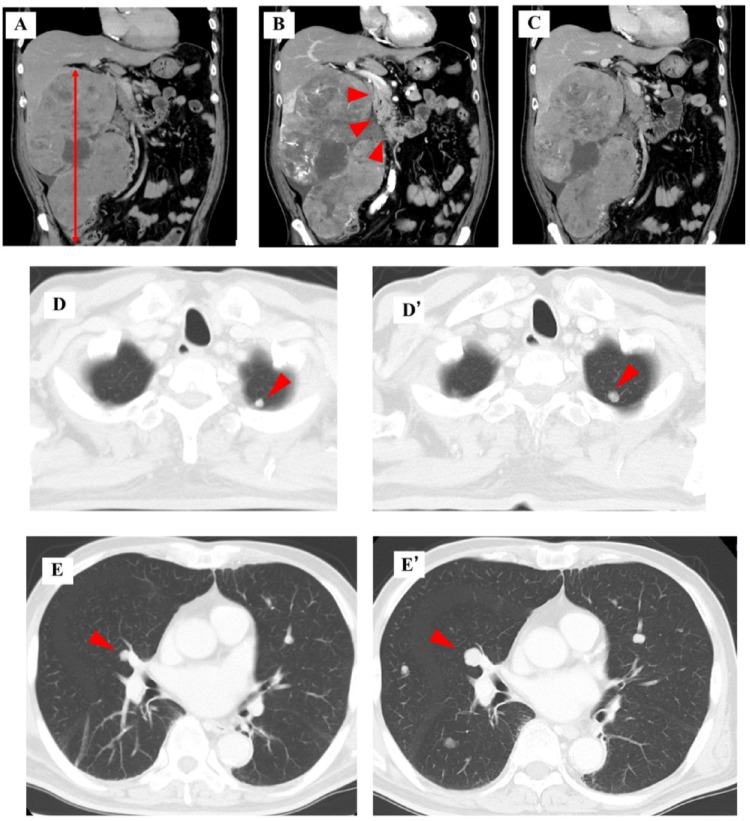
A large tumor of 270 mm in diameter (red arrow) occupying the right lobe of the liver on non-contrast CT (**A**), arterial-phase contrast-enhanced CT (**B**), and venous-phase contrast-enhanced CT (**C**). The tumor exhibits early enhancement and washout, suggesting hepatocellular carcinoma. The tumor was widely adherent to the ascending colon and duodenum (arrowheads). CT shows metastatic lesions in both lungs (arrowheads). CT of the lung nodules before (**D**, **E**) and after the hepatectomy (D’, E’). The lesions are indicated with arrowheads. Some lesions showed increase in size

An 80-year-old male presented to a local clinic in March 202X with complaints of abdominal distension and loss of appetite. Abdominal ultrasound detected a large mass, and the patient was referred to our hospital for further evaluation and treatment. On physical examination, a mass was palpated in the area extending from the right hypochondrium to the upper margin of the inguinal region. No edema was observed, but abdominal distension due to the mass was noted. His medical history included appendicitis treated surgically at the age of 50 and acute myocardial infarction at the age of 77. He had been undergoing pharmacological treatment for diabetes and hypertension. Blood tests revealed the following abnormalities in the biochemical panel: AST, 79 IU/L; LD, 479 IU/L; and CRP, 5.4 mg/dl. Complete blood count and coagulation tests showed elevated platelet count (36.1 × 10^4/µl) and increased D-dimer level (9.6 µg/dl). Serum alpha-fetoprotein (AFP) and des-γ-carboxy prothrombin (DCP) were both markedly elevated (AFP, 143,360 ng/ml; DCP, 18,962 mAu/ml). Regarding viral makers, HBc-Ab was positive, whereas HBs-Ag and HCV-Ab were negative. The indocyanine green retention rate at 15 min was 2.4%, and the disappearance rate was 0.1819 (Table 1). Contrast-enhanced CT identified a large hepatic mass with arterial-phase enhancement and portal-phase washout, supporting a diagnosis of HCC, despite the majority of the tumor protruding from the liver (Fig. 1A–C). The maximal tumor size was 270 mm in diameter, and it was compressing the ascending colon and duodenum. The tumor was in close contact with the hepatic hilum, including the right branch of the portal vein; however, there were no findings suggestive of portal vein tumor thrombosis. Chest CT revealed 15 round nodules, up to 10 mm in size, in the bilateral lungs (Fig. 1D, E). Gadoxetic acid-enhanced MRI also indicated solitary HCC and lung metastases, consistent with the contrast-enhanced CT findings. Endoscopic examination revealed external compression of the colonic wall; however, no lesions were noted on the mucosal surface.

**Table 1:** Preoperative blood test results

Variable	Result	Normal values
A, Blood count		
WBC, x103/µl	7.5	3.3–8.6
RBC, x106/µl	3.7	3.85–4.92
Hb, g/dl	10.6	11.6–14.8
Plt, x104/µl	36.1	15–40
B, Biochemistry		
T‑bil, mg/dl	1.0	0.2–1.2
D‑bil, mg/dl	0.42	0.0–0.3
AST, IU/l	79	8–30
ALT, IU/l	24	5–35
LD, IU/l	479	120–240
ALP, IU/l	110	100–340
γ-GTP, IU/l	56	10–30
TP, g/dl	6.2	6.5–8.2
Alb, g/dl	3.2	3.8–5.2
CRP, mg/dl	5.4	≤ 0.3
C, Coagulation		
APTT	26.9	25–35
PT, %	79.5	70–130
D-dimer, µg/dl	9.6	≤ 0.5
D, Tumor markers		
AFP, ng/ml	143,360	0–10
DCP, AU/ml	18,962	≤ 028

WBC, white blood cells; RBC, red blood cells; Hb, hemoglobin; Hct, Plt, platelet; T-bil, total bilirubin; D-bil, direct bilirubin; AST, aspartate aminotransferase; ALT, alanine aminotransferase; ALP, alkaline phosphatase; TP, total protein; Alb, albumin; CRP, C-reactive protein; APTT, activated partial thromboplastin time; PT, prothrombin time; AFP, α-fetoprotein; DCP, des-gamma-carboxy prothrombin

**Fig. 2:**
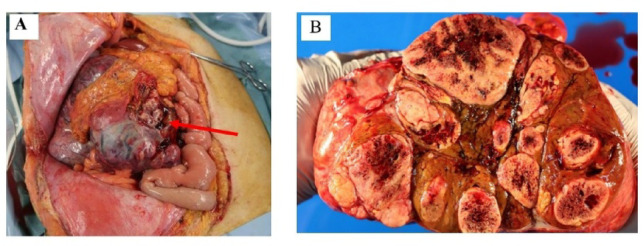
**A** Intraoperative view after laparotomy showing the ascending colon (arrows) being extensively compressed by the liver tumor. **B** Cut surface of the main liver tumor showing multinodular confluent appearance

**Fig. 3:**
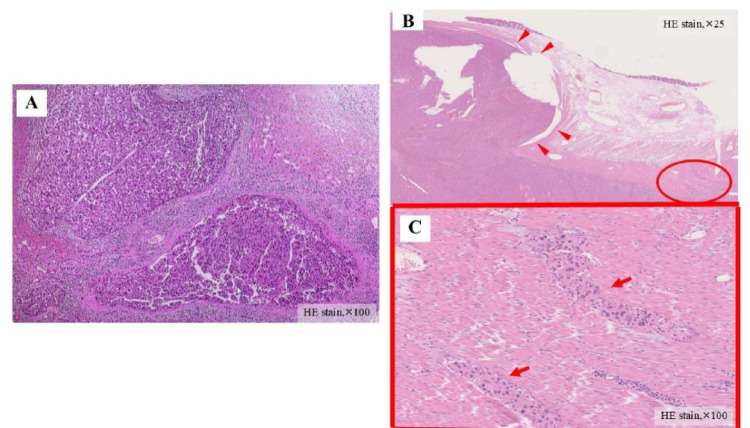
Microscopic imaging of the tumor. **A** Proliferating tumor cells with enlarged nuclei containing increased chromatin and eosinophilic cytoplasm. **B** shows hepatocellular carcinoma infiltrating the ascending colon. The red-highlighted area on the right indicates extrinsic compression of the colon by the tumor (red arrows). **C** is a magnified view of the red-circled area, demonstrating tumor cell infiltration into the muscular layer, as indicated by the red arrows

**Fig. 4:**
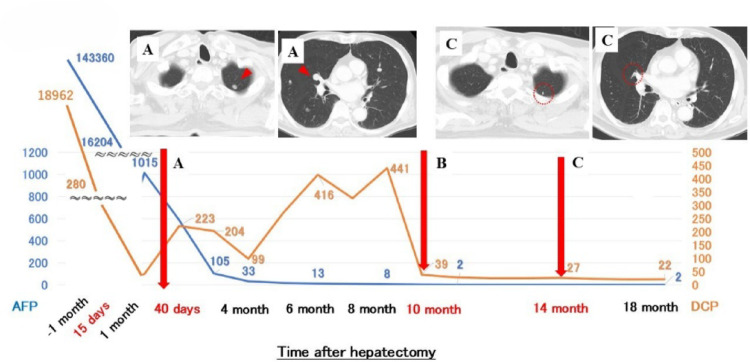
Postoperative course of the patient. **A** CT 40 days after surgery before the treatment with Atezolizumab + Bevacizumab showing multiple pulmonary metastases. **B** Ten months after surgery, the tumor markers are negative. **C** CT 14 months after hepatectomy showing remission of the pulmonary lesions

Based on the above tests, the preoperative diagnosis was HCC (cT4 cN0 cM1 [lung], cStage IVB) based on the 8th edition of the Union for International Cancer Control tumor-node-metastasis staging system [15]. According to current guidelines, systemic therapy, including combination immunotherapy with atezolizumab plus bevacizumab, was considered as the initial treatment for advanced hepatocellular carcinoma. However, at presentation, the patient already had abdominal distension and loss of appetite. Further evaluation revealed marked compression of the duodenum by the tumor, causing obstructive symptoms. In addition, the tumor was adjacent to the hepatic hilum, raising concern for sudden deterioration due to intratumoral hemorrhage or rupture. Although the patient was 80 years old, his performance status was preserved. Given the condition that oral intake was difficult, our multidisciplinary team recommended to perform surgical resection of the primary tumor first followed by systemic chemotherapy. To relieve abdominal distension and reduce the risk of tumor rupture, the primary tumor was resected. The procedure was performed via open laparotomy and consisted of right hepatectomy combined with right hemicolectomy. The duration of the surgery was 7 h and 50 min. Intraoperative blood loss was 4086 ml. Intraoperative findings revealed that the ascending colon was extensively compressed by the liver tumor, with strong adhesions (Fig. 2A). Therefore, combined resection of the cecum and ascending colon was performed. Postoperative pathological examination revealed that the lesion, which was preoperatively considered to be a single tumor, was the main tumor. The primary tumor was located in liver segments (S) 5/6, with an intrahepatic metastatic lesion in S6/7 distal to the portal vein tumor thrombus. The tumor in S5/6 protruded outside the liver and measured 252 mm in diameter, whereas the tumor in S6/7 measured 119 mm (Fig. 2B). Macroscopically, the cut surface of the tumors showed a multinodular confluent pattern. Microscopically, there was evidence of tumor cell proliferation with enlarged nuclei, increased chromatin, and eosinophilic cytoplasm, as well as the appearance of nucleoli. Mild infiltration of lymphocytes and plasma cells was also observed (Fig. 3A), as well as infiltration of HCC into the ascending colon (Fig. 3B, C). Based on these findings, the postoperative diagnosis was moderately to poorly differentiated HCC invading the ascending colon and omentum, with microscopic portal vein invasion (pT4 N0 M1 [lung], pStage IVB). The postoperative course was uneventful and there were no complications. On the 8th postoperative day, the patient was discharged. On the 15th postoperative day, AFP and DCP decreased to 16,204 ng/ml and 280 mAu/ml, respectively (Fig. 4). After confirming adequate wound healing, atezolizumab plus bevacizumab was initiated on postoperative day 40 to treat the 15 pre-existing pulmonary nodules identified on CT, which had shown a tendency to increase in size (Fig. 1D*’*,* E’*).

This timing was determined in consideration of the potential risks of impaired wound healing and bleeding associated with bevacizumab, and was consistent with guidelines and previous reports recommending the interval of at least 4–6 weeks after major surgery [16]. The treatment was administered every three weeks. Severe proteinuria was observed after 24 cycles, and bevacizumab was discontinued thereafter. A total of 29 cycles of chemotherapy were delivered, and the therapy was discontinued 33 months after the surgery. At this time, DCP was 441 mAu/ml; however, further tests 10 months after surgery showed normalized AFP (8 ng/ml) and DCP (39 mAu/ml). CT 14 months after hepatectomy showed no obvious recurrence in the abdomen, and all multiple lung metastases had almost disappeared leaving only scar-like lesions. Radiologic complete remission was achieved 14 months after hepatectomy, and both AFP and DCP were normalized. Totally, 29 courses of chemotherapy were performed; therefore, chemotherapy was discontinued in the 33rd postoperative month. As of 54 months after surgery, there has been no sign of tumor recurrence.

## Discussion

This case was unique in demonstrating pathological confirmation of direct colonic invasion by hepatocellular carcinoma and radiologically confirmed complete remission of pulmonary metastases. These findings suggest the feasibility of upfront hepatectomy followed by systemic chemotherapy for advanced HCC with synchronous extrahepatic metastases.

Direct invasion of HCC into adjacent organs is rare and to date only 16 cases of direct invasion of HCC to the adjacent colon have been reported in the English-language literature [17–25]. Among these cases, the most frequent symptom was hematochezia (9 out of 16 cases). All 16 cases presented with symptoms related to intestinal invasion such as epigastric pain, melena, or positive fecal occult blood tests. In the present case, although epigastric pain may have been masked by the abdominal distension, no symptoms related to intestinal invasion were observed. In some previously reported cases, colonic invasion was suspected on preoperative CT, and in patients with more severe symptoms, endoscopy revealed luminal stenosis due to tumor invasion. Among the 16 cases, eight had undergone transarterial embolization or transarterial chemoembolization (TACE) and colonic resection was performed in five cases. In some cases, only palliative colostomy was performed to relieve symptoms (Table 2). In the present case, the massive tumor was in broad contact with the ascending colon; however, no findings suggestive of bowel obstruction were observed. Therefore, we initially assumed that the colon was merely compressed by the tumor. Intraoperatively, the tumor was found to be tightly adherent to the colon, and direct invasion into the muscular layer of the colon was histologically confirmed.

**Table 2:** Summary of reported cases of colorectal metastasis from hepatocellular carcinoma

Study	Number of patients	Age (years)/sex	Symptoms	Mode of spread	Previous treatment for HCC	Treatment for colonic lesion	Follow-up (months)	Survival
Hashimoto et al. [17]	1	72/f	Bloody stool	Direct invasion	TAE	Operation	4	Yes
Chen et al. [18]	1	71/m	Bloody stool	Direct invasion	TAE	No	6	No
Lin et al. [19]	4	59–67/all m	Bloody stool (2), occult blood positivity (2)	Direct invasion	TAE (2)	No	1.2, 1.2, 1.5, 4.0	No
Srivastava et al. [20]	1	32/m	Bloody stool	Direct invasion	TAE	TAE	0.7	No
Zech et al. [21]	1	57/m	Abdominal pain	Direct invasion	TACE	Operation	Not available.	Not available.
Hirashita et al. [22]	2	79/m, 69/m	Abdominal pain, bloody stool	Direct invasion	TACE་ablation therapy	Operation	6, 1	No
Park et al. [23]	4	Mean, 58,all m	Not available.	Direct invasion, intraluminal spread	Not available.	Not available.	Not available.	Not available.
Nozaki et al. [24]	1	69/m	Abdominal pain་bloody stool	Direct invasion	Operation	No	1	No
Cosenza et al. [25]	1	82/f	Bloody stool	Direct invasion	Operation	Operation	25	Yes

f, female; m, male; TAE, Transcatheter Arterial Embolization; TACE, Transcatheter Arterial ChemoEmbolization

Previous studies have reported that hepatocellular carcinoma with adjacent organ invasion is associated with a poor prognosis, with postoperative survival ranging from 1 to 6 months [12, 13]. However, these previous studies did not specify whether postoperative chemotherapy was administered. The present case was notable regarding the achievement of long-term survival in a patient with highly advanced disease with extensive local spread and extrahepatic metastases.

The long-term survival observed in this case may have been attributable to the postoperative administration of atezolizumab plus bevacizumab. This is supported by the remarkable response of the pulmonary metastases to treatment, and the complete disappearance of the lung lesions represents a particularly novel and noteworthy finding.

However, the interpretation of tumor marker kinetics in this case requires careful consideration. While AFP showed a continuous and marked decline after surgery, it normalized only after the initiation of chemotherapy. In contrast, DCP demonstrated a transient elevation, which may not necessarily reflect tumor progression, as DCP can be influenced by nutritional status and vitamin K metabolism. Although the relatively monotonic decline of AFP after hepatectomy might raise the possibility that the pulmonary lesions were not metastatic HCC, this cannot be definitively concluded.

In the present case, multiple bilateral pulmonary nodules were observed in the setting of advanced HCC. These nodules appeared well-demarcated and were not typical of inflammatory lesions. Furthermore, all lesions showed slight size increase after the hepatectomy and marked radiological response to systemic therapy and eventually disappeared, leaving only scar-like remnants. Considering the overall clinical course—including the postoperative decline and subsequent normalization of AFP after chemotherapy initiation, the pulmonary lesions were considered most likely metastatic HCC, although the absence of pathological confirmation remains a limitation.

Previous studies of the successful administration of atezolizumab plus bevacizumab for the treatment of unresectable hepatocellular carcinoma have primarily reported cases in which initially unresectable tumors became amenable to surgical resection following a favorable treatment response. On the other hand, the role of systemic chemotherapy after reduction surgery, as was applied in the present case, is controversial [25, 26]. Satoh reported a patient with a giant HCC (13.5 cm) in the right hepatic lobe accompanied by multiple intrahepatic metastases (five lesions in the medial segment, two in the lateral segment) who underwent right hepatectomy, followed by postoperative TACE with epirubicin [27]. After the appearance of pulmonary metastases, TACE was switched to cisplatin (CDDP), followed by systemic chemotherapy with 5-fluorouracil and CDDP. Ultimately, sorafenib was introduced. The patient survived for 26 months postoperatively, with no progression of either pulmonary or intrahepatic metastases at the final evaluation. In advanced HCC cases in which the primary tumor occupies more than 50% of the liver volume, TACE alone is often insufficient for disease control. Satoh’s case report suggests that tumor burden reduction through debulking surgery followed by multidisciplinary treatment enables long-term disease control. Currently, for advanced HCC, a chemotherapy-first approach aiming conversion from unresectable to resectable disease has become a popular strategy. However, our findings suggest that reduction surgery followed by systemic chemotherapy to eradicate residual disease may also represent a reasonable therapeutic option with curative intent. In the present case, we performed upfront resection of the primary tumor because the patient was symptomatic and we considered that the extensive primary tumor rather than the pulmonary metastases would be the prognostic factor. Even in the patients with systemic disease, controlling the dominant lesions through cytoreductive surgery may reduce tumor burden, alleviate symptoms, and potentially enhance the efficacy of subsequent systemic therapy.

## Conclusion

In the era of effective systemic chemotherapy capable of converting initially unresectable hepatocellular carcinoma to resectable disease, upfront reductive surgery followed by postoperative systemic chemotherapy may represent a viable option for selected patients with extensive disease.

## Declarations

of 1975, as revised in 2008 (5).
